# Synthesis, Structural and Sensor Properties of Nanosized Mixed Oxides Based on In_2_O_3_ Particles

**DOI:** 10.3390/ijms24021570

**Published:** 2023-01-13

**Authors:** Mariya I. Ikim, Genrikh N. Gerasimov, Vladimir F. Gromov, Olusegun J. Ilegbusi, Leonid I. Trakhtenberg

**Affiliations:** 1 N.N. Semenov Federal Research Center for Chemical Physics of RAS, Moscow 119991, Russia; 2 Biomedical and Process Modeling Lab, University of Central Florida, Orlando, FL 32816, USA; 3 Moscow Institute of Physics and Technology, State University, Dolgoprudny 141701, Russia; 4 Chemical Faculty, Lomonosov Moscow State University, Moscow 119991, Russia

**Keywords:** tin oxide, indium oxide, nanocomposite, impregnation method, sensor response, conductivity

## Abstract

The paper considers the relationship between the structure and properties of nanostructured conductometric sensors based on binary mixtures of semiconductor oxides designed to detect reducing gases in the environment. The sensor effect in such systems is determined by the chemisorption of molecules on the surface of catalytically active particles and the transfer of chemisorbed products to electron-rich nanoparticles, where these products react with the analyzed gas. In this regard, the role is evaluated of the method of synthesizing the composites, the catalytic activity of metal oxides (CeO_2_, SnO_2_, ZnO), and the type of conductivity of metal oxides (Co_3_O_4_, ZrO_2_) in the sensor process. The effect of oxygen vacancies present in the composites on the performance characteristics is also considered. Particular attention is paid to the influence of the synthesis procedure for preparing sensitive layers based on CeO_2_–In_2_O_3_ on the structure of the resulting composites, as well as their conductive and sensor properties.

## 1. Introduction

Currently, the most promising method for determining the content of various substances in the environment is the use of sensors based on nanostructured semiconducting metal oxides. The operating principle of such sensors (see, for example, reviews [1,2,3]) is based on the chemisorption of oxygen and analyzed gases by a sensitive metal oxide layer, wherein, active oxygen particles are formed, such as O_2_^−^, O^−^ and O^2−^, which capture electrons from the conduction band of the metal. The nature of these centers depends on temperature [4]. Under real sensor operating conditions in air at temperatures of 200–400 °C, such centers are O^–^ anion radicals [1]. However, the data of Sun et al. [5] show that at 300 °C in a humid atmosphere, O^2−^ particles play a more important role in the sensory process involving indium oxide.

The analyzed gas adsorbed on the surface of the sensitive layer interacts with active oxygen centers. As a result of this reaction, the electrons captured by the anion radicals are released and passed into the conduction band of the oxide, thereby changing its conductivity. It is this change in the conductivity of the metal oxide sensor in the presence of analyzed gases that constitutes the sensor effect.

The efficiency of resistive sensors depends on many factors. The most important factor, the so-called receptor function, is the ability of the sensitive layer to adsorb gas molecules, which then react with the metal oxide molecules that make up this layer. The transformation function, i.e., the ability of a sensor system to convert the results of a chemical reaction between the metal oxide and gas molecules into an electrical signal [6,7], also plays an important role. All these factors are influenced by the chemical composition and structure of the sensor materials, which, in turn, depend on the method of their synthesis.

Unfortunately, the previously developed one-component semiconductor sensors based mainly on tin oxide (the so-called Taguchi sensors [8]) do not have sufficiently high sensitivity and selectivity with respect to compounds of the same type, for example, various reducing gases. Thus, in recent years, many research centers have been intensively investigating the sensor activity of two-component nanostructured semiconductor systems containing metal oxides of various types. The results of these studies have been summarized in several recently published reviews (see, for example, [3,9,10,11]).

The study of this problem has revealed a significant effect of the structure and composition of nanostructured binary metal oxide systems obtained by various methods on the conductivity and sensor properties of the oxide systems. It has been established that, in order to obtain highly efficient sensors, they must consist of oxides with different chemical and physical properties, in particular, high conductivity and catalytic activity.

One of the factors that determine the sensory properties of a metal oxide composite is the interaction between its components [1,10]. This interaction may be due to specific contacts between nanoparticles of various metal oxides present in the composite. As a result of these contacts, there occurs a transfer of atoms, molecules, and ions forming on the catalytically active nanoparticles during the dissociation of molecules of the studied gas and oxygen, to the nanoparticles that are rich in electrons [1,2,3,9,10,11,12,13]. The mutual charging of particles during the transfer of electrons between nanoparticles is also possible [14,15,16].

In addition, the interaction between metal oxides during the formation of a composite may involve doping, i.e., the introduction of metal ions of one oxide into the crystal lattice of another oxide. In this case, the ions are replaced in the crystal lattice of the particles, thereby forming a solid solution. Ultimately, this can have a significant impact on the sensor properties of the composite. 

The effect produced by the introduction of metal ions into the lattice of another oxide depends on the relative sizes and valence of metal ions in the components of the sensor system. A particularly strong influence on the sensor process is exerted by heterovalent doping, in which the valences of metal ions in the base and doping oxides differ from each other. In this case, during the formation of the composites, the insertion of ions of one metal oxide component into the nanoparticles of another causes a redistribution of charges in the system and thus affects its conductivity.

Depending on the properties of the metal oxides, their combination in a composite can have both positive and negative effects on sensor properties [12]. Therefore, to create new materials based on nanostructured metal oxides with a specific set of properties, it is necessary to determine the characteristics of the interaction between the components of the composite. In addition, it is important to establish the influence of this interaction, as well as the nature of the components and the method of formation of composites, on their sensory properties.

This article provides a brief overview of recent publications devoted to various factors affecting the characteristics of gas sensors based on metal oxide nanocomposites. The problems of modifying the sensitive layer are considered in order to develop the principles for improving the operational characteristics of semiconductor sensors such as the minimal response time, maximal sensor response, and minimal operating temperature. Special attention is paid to the insufficiently studied role of the methods of composite formation on their sensor properties. The effect of modifying additives on the characteristics of a metal oxide sensor can also change radically depending on the method of incorporating such additives into the sensitive layer. All these factors should contribute to further progress of the scientific and applied research necessary for the development of highly efficient sensors. Such sensors should be capable of timely and reliable early warning of the occurrence in the atmosphere of toxic, flammable, and explosive substances, as well as compounds that produce an epidemic hazard.

## 2. Factors Affecting the Efficiency of Sensitive Layers

The properties of mixed metal oxide composites can vary over a wide range depending on the composition of the composites, the method of their formation, and the nature of the components, particularly their conductivity and catalytic activity. The correct selection of these parameters creates new possibilities for the purposeful synthesis of materials with desired properties.

### 2.1. Influence of Catalytically Active Metal Oxide

The effect of additives of a catalytically active component to the matrix oxide, which provides the conductivity of the composite, on the properties of the composite depends on the nature of the matrix component. Such a picture is observed when considering the properties of composites obtained by the impregnation method and consisting of a matrix oxide, which provides the current flow paths in the composite and catalytically active cerium oxide [17]. In_2_O_3_ and SnO_2_, which have different conductivity, were used as the matrix oxide. Cerium oxide is an effective catalyst for the oxidation of organic compounds [18] and promotes the formation of chemisorption centers for oxygen molecules and various gases involved in sensor processes [19].

**Figure 1:**
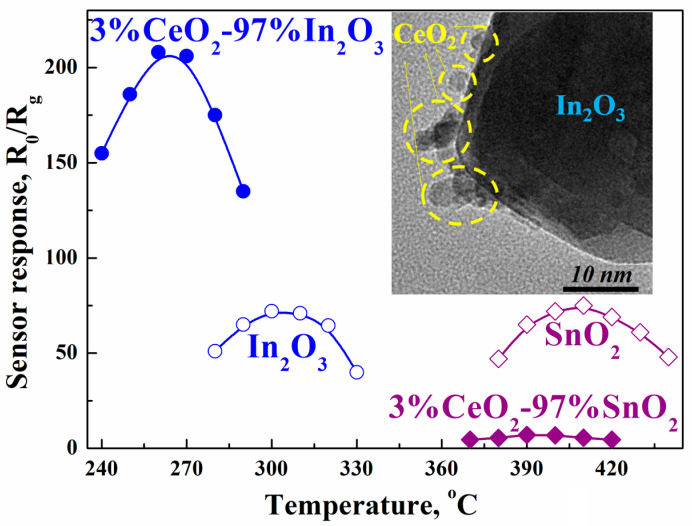
Temperature dependence of the sensor response of composites containing 3% CeO_2_ applied for detection of 1100 ppm H_2_. Inset: TEM image of 3%CeO_2_–97%In_2_O_3_ composite.

The preparation of these composites by the impregnation method in both cases leads to the formation of a structure consisting of matrix oxide nanoparticles of 50–70 nm in size, on the surface of which are small cerium oxide clusters, the size of which does not exceed 10 nm. At the same time, cerium oxide additives have different effects on the sensor properties of the prepared composites. When even small amounts (1–3 wt.%) of CeO_2_ are added to tin oxide, the sensor response of the composite to hydrogen drops sharply, and at 10 wt.% cerium oxide, this system becomes practically inactive. However, the addition of 10 wt.% CeO_2_ to indium oxide, whose conductivity is 3–4 orders of magnitude higher than that of tin oxide, leads to a sharp increase in the sensor response to hydrogen, which is approximately three times the response of pure indium oxide (Figure 1).

A detailed discussion of the properties of the CeO_2_–In_2_O_3_ system is given in Section 3, thus, the present section only discusses the influence of various factors on the behavior of the SnO_2_–CeO_2_ composite.

When considering the CeO_2_–SnO_2_ system, it should be noted that, unlike the CeO_2_–In_2_O_3_ system, CeO_2_ additives modify the structure of tin oxide, which provides the conductivity of the composite at low concentrations of CeO_2_. This modification is due to the dissolution of cerium ions in the SnO_2_ lattice. These data are consistent with the results of other studies [20,21,22,23,24,25,26]. The resulting modification of the SnO_2_ structure leads to a weakening of the Sn–O bond, as a result of which the mobility of oxygen anions participating in the sensor reaction increases and the rate of their desorption from the SnO_2_ surface increases.

An increase in the sensory activity of SnO_2_ is observed when it is doped with metal ions that differ from the valence of tin, in particular, zinc ions or trivalent metal ions [27,28,29]. Nanostructured SnO_2_-ZnO heterostructures are highly sensitive to CO [27]. Doping SnO_2_ with trivalent metal ions leads to a significant increase in the efficiency of tin oxide in low-temperature hydrogen detection [29].

A decrease in the concentration of oxygen anions produces a drop in the sensor effect. The observed deformation of the tin oxide lattice probably increases the depth of oxygen vacancies formed in this lattice. This assumption is confirmed by the results of measuring the electrical conductivity of the studied SnO_2_–CeO_2_ composites. In particular, the activation energy of conduction, determined by the depth of oxygen vacancies, in SnO_2_–CeO_2_ is 1.5–2 times that of SnO_2_ [12].

It has been established that the dependence of the sensor effect on the concentration of doping CeO_2_ ions is similar to the dependence on the amount of chemisorbed oxygen. The sensor effect (which is defined as the ratio of sample resistance in air to the resistance in the presence of analyzed gas) in the detection of reducing gases is determined by the chemisorption of oxygen and the analyzed gas on metal oxide particles and the reaction of chemisorbed gas molecules with oxygen ions. This finding indicates the effect of CeO_2_ on oxygen chemisorption.

### 2.2. Influence of the Synthesis Method of Metal Oxide Composites

The properties of binary metal oxide sensors largely depend on the method of their preparation. This is indicated, especially by the data on the conductivity and sensor properties of SnO_2_–In_2_O_3_ composites obtained from a mixture of nanopowders by screen printing [30,31,32,33], when In_2_O_3_ is impregnated with tin oxide [34,35,36], and by the hydrothermal method [37,38]. Composites formed by these methods contain nanoparticles of various sizes. If in the first case, the particle size of both oxides is 50–70 nm, then the impregnated composite consists of In_2_O_3_ matrix oxide nanoparticles (about 50 nm in size), on the surface of which there are SnO_2_ nanoclusters 5–7 nm in size. The hydrothermal composite consists of particles of approximately the same size (about 10–20 nm).

**Figure 2:**
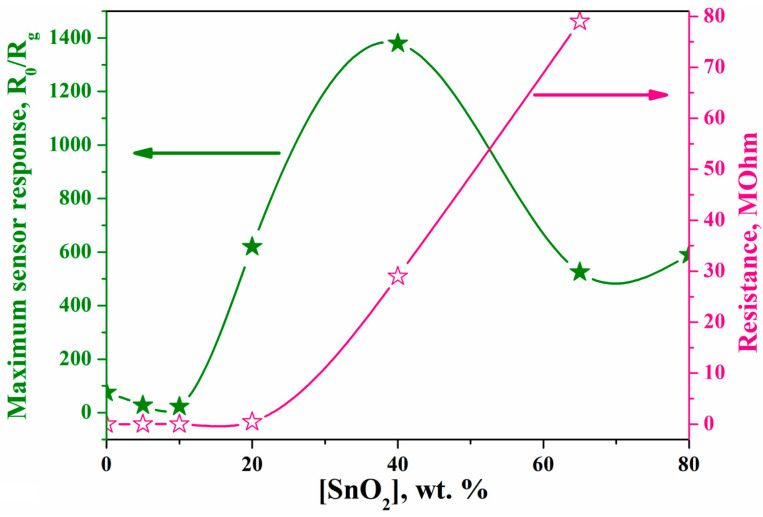
Dependence of the maximum sensor response at 1100 ppm H_2_ (green stars) and the resistance of the SnO_2_–In_2_O_3_ composite (red stars) on the content of tin oxide.

The addition of small amounts of SnO_2_ up to 7–8 wt.% leads to a drop in the resistance and sensory response of the impregnated composite [34,35]. With a subsequent increase in the amount of SnO_2_, both the resistance of the composite and the detection efficiency increase. The high sensory sensitivity of the composite is explained by the catalytic activity of SnO_2_ nanoclusters containing indium ions and their high specific surface area, which ensures the conductivity of the system (Figure 2).

The maximum sensor response of the impregnated composite to 1100 ppm of hydrogen at 320 °C, which is 1400, is achieved at a content of 40 wt.% SnO_2_. In such a composite, according to the percolation theory, the current flow paths are aggregates of SnO_2_ nanoclusters contacting with In_2_O_3_ crystals. Characteristically, the maximum response is observed when the SnO_2_ clusters contain the maximum amount of dissolved indium ions.

The dissolution of In_2_O_3_ in the lattice of the SnO_2_ cluster and the replacement of Sn^+4^ ions by In^+3^ ions leads to the formation of local negative charges, which are compensated by positively charged oxygen vacancies [39]. Such a change in the structure of the composite contributes to an increase in its sensor activity. The interaction of oxygen vacancies with gas molecules depends on the structure and location of the vacancy [40]. The high sensor response is also due to electron transfer from In_2_O_3_ nanoparticles to SnO_2_ nanoclusters.

A further increase in the SnO_2_ content to 60–65 wt.% produces a sharp drop in sensor activity due to a decrease in the number of oxygen vacancies resulting from the removal of indium ions from the lattice of SnO_2_ clusters. This is evidenced by a decrease in interplanar spacing d(110) in SnO_2_ clusters with increase in the SnO_2_ concentration in the composite to 65 wt.% [35]. In this case, the structure of these clusters in the composite approaches the structure of the initial SnO_2_ clusters.

In contrast to the impregnated system for composites obtained by mixing oxide nanopowders, the maximum response to hydrogen, which is 1.5 times than the response of pure tin oxide sensor, is achieved at a content of 80 wt.% SnO_2_ [31]. Concurrently, the minimum response occurs in the composite containing 50 wt.% tin oxides. In the range from 0 to 50 wt.% SnO_2_, only a slight drop in sensor activity is observed.

Indium atoms are not introduced into the SnO_2_ lattice in composites obtained by mixing nanopowders. Therefore, the observed effect of composition on the sensor effect is due to a change in the conduction paths in systems with different content of In_2_O_3_ [30,31,32,33]. When the content of SnO_2_ is more than 80 wt.%, the current flows through the SnO_2_ crystals. Additives of In_2_O_3_ have a significant effect on the conductivity of SnO_2_ in a composite film.

The temperature dependence of the conductivity of SnO_2_–In_2_O_3_ composite films containing In_2_O_3_ ≥19 wt.% indicates the flow of current through an endless conducting cluster of In_2_O_3_ nanocrystals. In the high-temperature range from 380 to 500 °C (when, probably, the complete replacement of molecular chemisorbed anions by atomic O^−^ anions occurred), the conductivities of both pure nanocrystalline In_2_O_3_ and a composite nanocrystalline SnO_2_–In_2_O_3_ film containing 50% indium oxide are nearly independent of temperature. At the same time, the conductivity of films containing 19 wt.% and 37 wt.% In_2_O_3_ noticeably increases with temperature in the indicated temperature range. This effect is much more pronounced in the film with 19 wt.% In_2_O_3_ than in the film with 37 wt.% In_2_O_3_. It should be noted that the temperature dependence of the conductivity in these films deviates from the Arrhenius dependence [30]. The data obtained indicate that the SnO_2_ nanocrystalline matrix affects the conductivity of a cluster of In_2_O_3_ nanocrystals located in the matrix.

In the presence of In_2_O_3_ nanocrystals, the activation energy of electron transfer between SnO_2_ nanocrystals noticeably decreases. Due to the transfer of electrons from In_2_O_3_ to SnO_2_, an increase in the content of indium oxide (X_In_) to 20 wt.% leads to a significant increase in the conductivity of the composite sensor and a change in sensor activity when detecting both CO and H_2_. According to the data on the conductivity of such composites, as X_In_ increases beyond 20 wt.%, infinite clusters of interconnected In_2_O_3_ particles are formed in the system, and the threshold for current flow through these particles is overcome.

The conductivity of In_2_O_3_ clusters and their sensitivity to CO significantly exceed the conductivity and sensitivity of tin dioxide to CO, so that at X_In_ ≥ 20 wt.%, the sensor properties of these clusters determine the sensor properties of the SnO_2_–In_2_O_3_ composite. A complete transition to conduction through In_2_O_3_ clusters occurs at X_In_ ≥ 50 wt.% [31]. Similar results were also observed in the TiO_2_ + In_2_O_3_ composite [41].

It should be noted that the response to hydrogen of impregnated composites containing 65 and 80% SnO_2_ nearly coincides with the response of the sensor obtained by mixing nanocrystalline metal oxides. The main factor affecting the response of the sensor is the transfer of electrons from In_2_O_3_ to SnO_2_. The interaction of hydrogen with chemisorbed oxygen leads to an increase in the difference (ΔW) between the work functions for SnO_2_ and In_2_O_3_, which is accompanied by sensitization of the electron transfer process. An increase in the value of ΔW promotes the flow of current along the conduction paths through the SnO_2_ nanoclusters due to the contribution of the conduction electrons of the In_2_O_3_ nanocrystals in contact with the SnO_2_ nanoclusters, which enhances the sensor response [35].

The results of X-ray diffraction showed that the 90% In_2_O_3_–10% SnO_2_ composite formed by the hydrothermal method consists of three phases: In_2_O_3_, SnO_2_, and In_2_Sn_2_O_7−x_ (mixed indium-tin oxide: ITO). The maximum response of this composite to 200 ppm CO is 9.2, which is among the highest values for CO sensors published over the past 10 years. The sensor response to CO of such a composite is more than four times the response to reducing compounds like benzene, toluene, and acetone [37].

**Figure 3:**
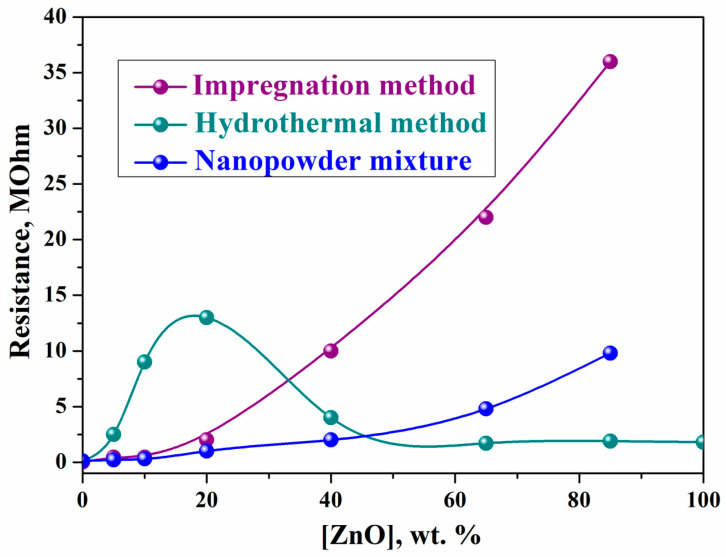
Dependence of the resistance of the ZnO–In_2_O_3_ composites synthesized by different methods on the content of zinc oxide.

The method of synthesizing the composite systems also affects the structure and conductivity of the ZnO–In_2_O_3_ system (Figure 3). The composites investigated were prepared using three different methods: mixing oxides by screen printing [42]; impregnating In_2_O_3_ crystals with zinc oxide; and the hydrothermal method.

**Figure 4:**
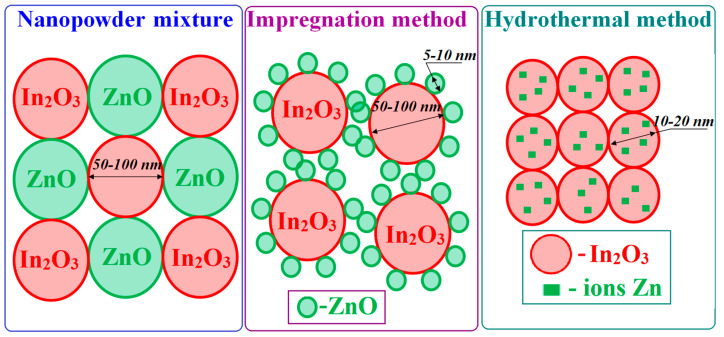
The schematic representation of ZnO–In_2_O_3_ composites structure synthesized by different methods.

To obtain hydrothermal composites, a NaOH solution was added to a mixture of indium and zinc nitrate salts. The resulting precipitate of the mixture of oxides was washed with water to remove sodium nitrate, then placed in a stainless-steel autoclave with a Teflon coating and kept for 1 h at 180 °C. After that, the resulting mixture was calcined at 500 °C for 3 h in air to transform into ZnO–In_2_O_3_ metal oxide nanocomposites. Note that composites with a ZnO concentration exceeding its solubility in In_2_O_3_ (about 3 wt.%) are conglomerates of interacting In_2_O_3_ and ZnO nanocrystals. Figure 4 schematically shows the structure of composites synthesized by different methods. 

According to XRD data, the impregnation method leads to the formation of only two-phase systems ZnO–In_2_O_3_ at different content of zinc oxide in the composite. In contrast to impregnated samples, in hydrothermal composites with a content of up to 20% zinc oxide, ZnO particles are not formed, and zinc ions are introduced into the indium oxide matrix. This leads to a modification of the electronic structure of In_2_O_3_ and a decrease in the concentration of conduction electrons, which increases the resistance of the nanocrystalline In_2_O_3_.

The incorporation of zinc into the structure of indium oxide promotes the formation of *p*-type defects and, consequently, increases the resistance of hydrothermal samples containing up to 20 wt.% zinc oxide. When the mixture contains more than 20 wt.% zinc oxide, ZnO crystals also appear during the formation of the composite. When these crystals come into contact with modified In_2_O_3_ crystals, the resistance of the films sharply decreases.

The specific surface area of the samples formed by various methods is 25 m^2^/g for the hydrothermal sample and 5 m^2^/g for the impregnated sample. The higher specific surface of the hydrothermal sample compared to the impregnated composite and the smaller average pore size (19 nm) facilitate the adsorption of oxygen and analyzed gas molecules on the surface of the hydrothermal sample. This adsorption contributes to the improvement in the dynamic characteristics of the sample, namely, an increase in the rate of response-recovery processes during detection application.

The addition of even small amounts of In_2_O_3_ to zinc oxide leads to a noticeable decrease in the resistance of the composite film obtained by mixing oxide nanopowders by screen printing [42]. In mixed composites with a low content of In_2_O_3_, current flows through the ZnO crystals, and the observed effect is due to an increase in the concentration of conduction electrons in ZnO because of electron transfer from In_2_O_3_ to ZnO. Concurrently, the addition of only 3 wt.% ZnO to the In_2_O_3_ film leads to an increase in the film resistance by approximately a factor of four and is accompanied by a noticeable increase in the response to hydrogen and CO. These effects are due to the modification of the electronic structure of In_2_O_3_ crystals as a result of their interaction with zinc oxide crystals. According to Kulkarni et al. [43], an increase in the resistance of In_2_O_3_ films doped with zinc oxide can also be associated with the effect of ZnO on the mobility of charge carriers due to their dispersion at the grain boundary of In_2_O_3_ and ZnO.

The modification of In_2_O_3_ with ZnO with the consequent formation of a heterojunction at the interface between nanoparticles, which promotes electron transfer to zinc oxide, not only increases the sensor response to formaldehyde, but also makes it possible to lower the limit of its detection [44].

The character of the dependence of conductivity on the composition of composites obtained by screen printing differs little from that of impregnated composites. In both cases, a monotonic decrease in conductivity is observed with increasing zinc oxide concentration. In contrast to these systems, the resistance of the hydrothermal sample reaches its maximum at 20 wt.% ZnO.

Addition of ZnO also leads to a significant increase in the sensor activity of SnO_2_. The sensitizing effect of zinc oxide made it possible to determine H_2_S in air at a concentration of 10 ppb and a working temperature of 100 °C [45].

### 2.3. Composites Based on Metal Oxides with Different Types of Conductivity

In order to elucidate the effect of the nature of metal oxides on the sensor process, the conductivity and sensor effect have been studied [46,47] in systems containing In_2_O_3_ having the conductivity of *n*-type and nanoparticles of metal oxide with a different type of conductivity, namely Co_3_O_4_ and zirconium oxide [46]. Cobalt oxide has hole conductivity, i.e., it is a *p*-type conductor; in ZrO_2_ at temperatures of 200–500 °C, there are practically no free electrons and, accordingly, there is no electronic conductivity.

The nature of the conductivity of the Co_3_O_4_–In_2_O_3_ system depends on its composition. At a cobalt oxide content of about 60 wt.%, there is a transition from electronic conduction through In_2_O_3_ nanocrystals to hole (*p*-type) conduction through Co_3_O_4_ aggregates. An increase in the content of cobalt oxide in the composite leads to a decrease in its conductivity. The most dramatic change is observed in the range from 10 to 60 wt.% Co_3_O_4_, when the resistance of the composite increases approximately 30 times and reaches more than 15 MΩ (see Table 1).

This effect is due to the interaction between the In_2_O_3_ nanocrystals that make up the current conduction paths in the composite and inclusions of the Co_3_O_4_ nanocrystals. Since the electron affinity of In_2_O_3_ (3.7 eV) [48] is much lower than that of Co_3_O_4_ (4.8 eV) [49], there is a transition of electrons from In_2_O_3_ to Co_3_O_4_, while the concentration of conduction electrons in In_2_O_3_ decreases, which leads to an increase in resistance.

Zirconium oxide has virtually no electronic conductivity and the current in the composite flows through “endless clusters” of In_2_O_3_ nanocrystals that penetrate the entire volume of the sample [50]. An increase in the ZrO_2_ content leads to a sharp decrease in the conductivity of the composite. This behavior is because ZrO_2_ additives catalyze the O_2_ dissociation reaction [51]. Since ZrO_2_ is an electron trap, oxygen atoms formed due to O_2_ dissociation on the ZrO_2_ surface transfer to In_2_O_3_ nanocrystals, where they capture conduction electrons, lowering their concentration in the conducting paths of the composite and thereby reducing the conductivity.

The addition of small amounts of Co_3_O_4_ to the In_2_O_3_ film, which does not change the *n*-type conductivity of the composite, results in a sharp increase in the response to hydrogen. The maximum response to hydrogen observed for a film containing 10 wt.% Co_3_O_4_ is greater than the response of pure In_2_O_3_ and Co_3_O_4_ (see Table 1) [46]. Such a change in the response is probably due to a change in the electronic interaction between Co_3_O_4_ and In_2_O_3_ under the influence of H_2_. Hydrogen reduces Co_3_O_4_ faster and easier than In_2_O_3_; therefore, in the presence of H_2_, part of the conduction electrons captured in air by Co_3_O_4_ particles returns to In_2_O_3_, and the conductivity of the composite increases. However, with an increase in the content of cerium oxide in the composite above 10 wt.%, the value of the response decreases.

The composite with 10 wt.% Co_3_O_4_ also has the maximum response to CO [46]. In this case, in addition to the above reasons, to explain sensitization, one should consider the fact that *p*-*n* contacts with electron transfer between *n*-type In_2_O_3_ nanocrystals and *p*-type Co_3_O_4_ nanoparticles are centers of preferential chemisorption of CO molecules. These centers have both electron-donating and electron-withdrawing properties. Thus, the addition of Co_3_O_4_ nanoparticles promotes an increase in the concentration of chemisorbed CO molecules and thereby increases the rate of the sensory response.

A sharp effect of cobalt oxide additions on the sensor effect was also observed in the SnO_2_–Co_3_O_4_ system [52,53,54]. However, for SnO_2_–Co_3_O_4_ composite nanofibers, in contrast to systems based on indium oxide, the maximum effect in the detection of CO, benzene, acetone, and SO_2_ was observed in a composite containing the same amount of tin and cobalt oxides [55].

**Figure 5:**
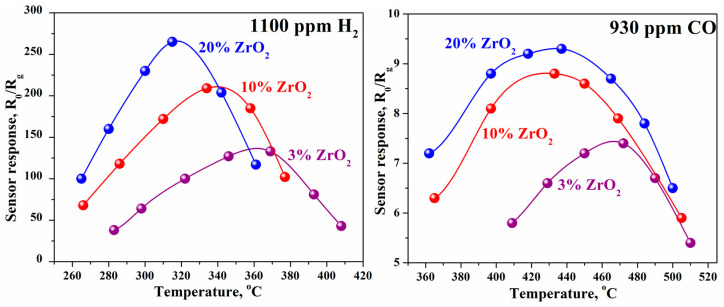
Temperature dependences of the sensor response of ZrO_2_–In_2_O_3_ composites at detection of 1100 ppm H_2_ and 930 ppm CO.

The sensor response of ZrO_2_–In_2_O_3_ in the detection of H_2_ and CO was studied for composites containing up to 20 wt.% ZrO_2_ [43]. In the range of compositions studied, the composite behaves like an *n*-type semiconductor. An increase in the ZrO_2_ content from 3 to 20 wt.% leads to an increase in the maximum response to 1100 ppm H_2_ from 120 to 280 and a decrease in maximum temperature, T_max_, from 370 to 315 °C (Figure 5). Addition of ZrO_2_ increases sensor activity in CO detection as well. However, in this case, the absolute value of the response is much smaller than for hydrogen. The maximum response to 930 ppm CO for composites containing 10 and 20 wt.% ZrO_2_ at 430 °C is 9.0–9.5.

The maximum sensor response to H_2_ noticeably increases with an increase in the ZrO_2_ content in the composite. At the same time, the response to CO when ZrO_2_ is added to In_2_O_3_ remains nearly invariant with a change in the composite composition. The difference in the effectiveness of ZrO_2_ additives in the detection of H_2_ and CO is because there are practically no free electrons in ZrO_2_ at temperatures of 200–500 °C and, accordingly, there is no electronic interaction between indium and zirconium oxides. Nevertheless, ZrO_2_, being a sufficiently strong catalyst, promotes the dissociation of oxygen and hydrogen molecules, leading to the formation of highly active particles, and thereby increases the rate of sensor reaction between these particles and the analyzed gas. Conversely, only the dissociation of oxygen occurs when detecting CO.

The effect of adding a *p*-type semiconductor to an *n*-type metal oxide on the sensor effect was also observed in the *p*-CuO + *n*-ZnO system [56]. The addition of 5% CuO to zinc oxide leads to an increase in the sensor response to ethanol by a factor of 4 compared to the response of pure ZnO. The main reason for this change in sensory activity is the formation of *p*-*n* heterojunctions upon contact of ZnO with CuO.

A change in the composition of a composite consisting of *n*-and *p*-type metal oxides is accompanied by a change in the type of conductivity. The response of the ZnO–0.425NiO composite annealed at 550 °C also depends on the temperature at which the detection process occurs. Thus, at an operating temperature of 300 °C, an *n*-type response is observed both for CO and H_2_; at 400 °C, the response for both gases is a *p*-type [57]. At the same time, at 350 °C, the composite exhibits an *n*-type response to H_2_ and a *p*-type response to CO. A change in the annealing temperature of the composite also affects the nature of the response: at 500 °C for both gases, a *p*-type response is observed, and at 600 °C, an *n*-type response. Thus, by changing the composition of such a composite, as well as the annealing temperature during its formation and the operating temperature during detection, it is possible to influence the selectivity of the composite in the detection of gases of the same nature. 

A similar behavior of composites consisting of oxides with *n-* and *p*-types of conductivity was also observed in the *n*-SnO_2_/*p*-xCuO system (here x is the ratio of CuO to SnO_2_ in composite) [58]. The nature of the response of these nanocomposites to H_2_ and CO depends on the content of *p*- and *n*-type oxides in them. The transition from *n*- to *p*-type response occurs at x = 2.78. A composite of this composition has the optimal sensitivity and selectivity in the detection of CO and H_2_: the responses to 400 ppm CO and H_2_ are 203.44 and 57.99, respectively. In *n*-SnO_2_/*p*-xCuO composites with *n*-type conductivity, an increase in the CuO concentration leads to an increase in the response to CO and H_2_. At the same time, in composites with *p*-type conductivity (at x > 2.78), the response value only slightly changes with increasing CuO concentration. This behavior may be due to the formation of numerous *p*-*n* junctions in the system, as well as a large amount of chemisorbed oxygen on the surface of the composite nanoparticles 6 nm in diameter.

A similar effect of the composition of the composite, its heat treatment, and operating temperature on the type of response and selectivity in the detection of H_2_ and CO was also established for a system containing SnO_2_ and Mn_3_O_4_ [59]. Nickel doping of SnO_2_ leads to an improvement in the sensor activity of the composite towards ethanol and its excellent stability at operating temperature. The response of Ni-substituted SnO_2_ to 1 ppm of ethanol was approximately 3 times than the response of pure SnO_2_ microspheres. XPS and O_2_-TPD spectroscopy data show that the observed increase in the sensitivity of the composite is due to an increase in the concentration of surface oxygen vacancies and, accordingly, the amount of chemisorbed oxygen [60].

Particular attention is drawn to the results obtained by using a mixture of two *p*-type oxides for doping an *n*-type metal oxide. A significantly higher sensitizing effect is observed when In_2_O_3_ is jointly doped with nickel and palladium oxides [61]. The co-doped PdO/NiO–In_2_O_3_ composite not only outperforms the response of pure indium oxide, but also results in a marked reduction in sensor operating temperature. By its characteristics, this composite surpasses the properties of known hydrogen sensors doped with only one metal oxide. Indeed, the response of the PdO/NiO–In_2_O_3_ composite to 5 ppm of hydrogen at 160 °C is 487.52, which is many times than that of such composites as NiO–In_2_O_3_ (2.48 at 300 °C) and PdO–In_2_O_3_ (16.21 at 160 °C). 

The use of a co-doped composite also leads to a sharp decrease in the response time to 1 s (l57 s for a sensor based on pure In_2_O_3_) and a slight decrease in the recovery time (168 s instead of 222 s for In_2_O_3_). The main reason for the observed effect of co-doping with *p*-type oxides is the formation of heterojunctions upon the contact of the nanocrystals.

### 2.4. Oxygen Vacancies

Oxygen vacancies play a decisive role in the characteristics of chemoresistive sensors based on metal oxides. The development of simple and efficient strategies to produce metal oxides with a controlled number of oxygen vacancies has attracted much attention and remains a top priority. The presence of highly concentrated oxygen vacancies narrows the band gap of semiconductors, thereby reducing the energy required for an electron transition. They also increase the number of active sites on the surface of the material and enhance chemisorption and thus, improve the adsorption characteristics of the material. Studies on the effect of concentration of oxygen vacancies on the sensor properties of semiconductor metal oxide materials have been reviewed [62,63].

The nature of oxygen vacancies that appear in the sensor material during formation is affected by the atmosphere in which it was calcined. During the calcination of SnO_2_ in air and in a helium atmosphere, materials with various types of defects are obtained [64]. When calcined in air, the predominant oxygen vacancy is Vo^••^. At the same time, when SnO_2_ is calcined in a helium atmosphere, triple associates of vacancies V_Sn_^////^V_o_^••^V_Sn_^////^ predominate. The nature of the vacancies has a noticeable effect on the sensory activity and selectivity of the sensor. The response to 100 ppm of ethanol and formaldehyde is 103 and 20.5 for samples containing V_o_^••^ vacancies and V_Sn_^////^V_o_^••^V_Sn_^////^ triple associates, respectively.

In the previous sections, some studies were presented that considered the role of oxygen vacancies in increasing the chemisorption of oxygen and analyzed gases (see, for example, [12,35,60]). In [65], a new method was proposed for obtaining SnO_2_ with a controlled content of surface oxygen. To obtain a modified product, toluene and (CH_3_)_2_SnCl_2_ were added to SnO_2_, heated in vacuum at 70 °C for 12 h, stirred for 30 min, and then a small amount of triethylamine was added. The solid product was separated in a centrifuge, washed with toluene and ethanol, dried for 12 h at 60 °C, and calcined for 3 h at 550 °C.

The product obtained as a result of such treatment contains 37.13% of oxygen vacancies on the surface, which is significantly higher than in untreated tin oxide (28.66%). The modified SnO_2_–Sn-0.5 sample, synthesized by adding 0.5 g of dimethyltin dichloride to 2 g of SnO_2_, had a high sensor activity in the detection of acetone. The sensory response to 100 ppm of acetone is 19.95 at 220 °C and is significantly higher than the response of commercial SnO_2_ under the same conditions (14.33). However, an increase in temperature to 270 °C led to a decrease in the response to 6.41 [65]. The response to 100 ppm acetone of sensors based on SnO_2_–Sn-0.25 and SnO_2_–Sn-0.75 synthesized by adding 0.25 and 0.75 g of (CH_3_)_2_SnCl_2_ to 2 g of SnO_2_ was 7.80 and 7.60, respectively. As indicated in [65], the slightly higher response of SnO_2_–Sn-0.25 is because the concentration of oxygen vacancies in SnO_2_–Sn-0.25 is lower than that of SnO_2_–Sn-0.75. This is due to the higher concentration of chemisorbed oxygen in SnO_2_–Sn-0.25 compared to SnO_2_–Sn-0.75.

Thus, the study of the sensor activity of the binary metal oxide systems considered above showed that they have high efficiency, selectivity, and an exceptionally high response rate to the analyzed gas when detecting toxic, flammable, and explosive gases in air such as hydrogen, CO, methane, ammonia, and ozone, which not only have reduced, but also oxidizing properties. The ability of these sensor materials to respond to the appearance of toxic, explosive, and flammable compounds in the atmosphere in concentrations exceeding the maximum permissible concentration (MPC) allows the use of these materials as sensors to signal violation of chemical safety.

## 3. Sensitive Layers Based on CeO_2_–In_2_O_3_

The method of preparing the sensitive layer also plays an important role to increase the efficiency of sensors along with the need for the correct selection of components. In this section, three different methods of preparing a sensitive layer are considered in detail, with focus on the CeO_2_–In_2_O_3_ system. These methods allow materials with different sensitivities to be obtained and makes it possible to understand the influence of the sensor preparation approach.

The choice of the components of the CeO_2_–In_2_O_3_ system is because indium oxide has the highest conductivity among metal oxides commonly used to obtain sensor materials, as well as a number of other practically important characteristics [66]. At the same time, CeO_2_ is a material with a direct band gap of 3.2 eV and excellent catalytic activity in reactions such as the oxidation of organic compounds. In addition, the structure of cerium oxide and its properties largely depend on the particle size: as the size of CeO_2_ nanoparticles decreases, the concentration of oxygen vacancies in them increases, and can lead to an increase in the catalytic activity of the particles [67]. This allows the use of cerium oxide in various fields of application [68,69,70,71]. The high concentration of oxygen vacancies, high thermal stability, and low redox potential of the Ce^4+^ ↔ Ce^3+^ transitions [72,73] have made cerium oxide desirable as a component in mixed metal oxide sensors, not only based on In_2_O_3_ [74,75], but also in combination with oxides of tin [23,25,76,77] or zinc [78]. Table 2 shows the values of the sensor response to reducing gases of binary composites containing CeO_2_ synthesized by various methods.

The formation of nanostructured CeO_2_–In_2_O_3_ composites was carried out in three different ways: (a) mixing pre-prepared nanopowders of indium and cerium oxides using the screen-printing method; (b) impregnation of In_2_O_3_ nanopowder with cerium oxide; and (c) hydrothermal method.

### 3.1. Mixing Nanopowders of Indium and Cerium Oxide

To form samples of sensitive elements, an aqueous suspension containing various concentrations of In_2_O_3_ and CeO_2_ powders with a nanoparticle size of ~50–70 nm was prepared. The resulting paste was applied by screen printing onto a dielectric substrate, on the reverse side of which were platinum contacts and a heater. The paste was dried for 3 h at 120 °C and then annealed in air, gradually raising the temperature to 550 °C. The resulting solid nanocrystalline film was kept at this temperature until a constant resistance value was reached. The film thickness averaged ~1 µm [17].

XRD data show that only In_2_O_3_ crystals are present in composites containing less than 20 wt.% CeO_2_ (6.5 at.% Ce). The lattice of the crystals coincides with the lattice of the cubic phase of In_2_O_3_. Peaks characterizing CeO_2_ crystals appear on the X-ray diffraction pattern only at higher concentrations of cerium oxide in the composite, and the lattice of these crystals coincides with the lattice of pure CeO_2_ [17].

The conductivity and sensor properties of the composite for the detection of H_2_ and CO depend on its composition. With an increase in the content of CeO_2_ (X_Ce_) from 0 to 40 wt.%, the resistance of the film increases from 4 to 24 kOhm. However, with further growth of X_Ce_, the resistance of the film sharply increases and, at 60 wt.% X_Ce_, it reaches 46 Mohm. Obviously, at X_Ce_ ≤ 40 wt.%, the current in the films passes through the aggregates of In_2_O_3_ nanocrystals, and the film conductivity is mainly determined by the electronic characteristics of the nanocrystals. 

An increase in the resistance of the In_2_O_3_ film when CeO_2_ is introduced into the film indicates that cerium oxide in these composites does not dissolve in In_2_O_3_ crystals. This conclusion is because the replacement of In^3+^ ions in the In_2_O_3_ lattice by Ce^4+^ ions (which occurs upon dissolution of CeO_2_ in the crystal lattice of indium oxide) leads to a sharp decrease in resistance [79]. When the content of cerium oxide in the composite is less than 20 wt.%, CeO_2_ does not dissolve in In_2_O_3_. Therefore, the structure of the CeO_2_–In_2_O_3_ composite comprises In_2_O_3_ nanocrystals, on the surface of which small CeO_2_ clusters are distributed [17].

The value of the sensor response of the composite to H_2_ and CO is determined by the temperature and composition of the composite. Addition of CeO_2_ increases the maximum sensor response of the composite, S_max_, and lowers the temperature, T_max_, at which the maximum response is observed. With an increase in the concentration of cerium oxide, the maximum response in the detection of these gases is observed at a content of 3–5 wt.% CeO_2_ in the composite. In this case, the response to H_2_ is approximately four times the response to CO.

The increase in the sensor response of the composite to hydrogen in the presence of small additions of cerium oxide is due to the promotion of dissociation of oxygen and hydrogen by CeO_2_ nanoparticles. These nanoparticles contain a high concentration of active oxygen vacancies, which are the centers of chemisorption of molecules. Oxygen and hydrogen atoms formed as a result of chemisorption on the surface of CeO_2_ nanoparticles are relatively weakly bound to their surface [19] and at temperatures in the range of 300–500 °C, they spill over on the surface of In_2_O_3_ crystal nanoparticles. The reaction of the hydrogen atoms with O^−^ anions, formed during the capture of electrons from the volume of In_2_O_3_ nanoparticles by oxygen atoms adsorbed on their surface, occurs at the interface between In_2_O_3_ and CeO_2_ nanoparticles. 

Similarly, the reaction of chemisorbed CO with O^−^ anions occurs at the interface between In_2_O_3_ and CeO_2_ nanoparticles. An increase in the rate of this reaction in combination with the diffusion of oxygen chemisorbed on the crystals to the reaction sites leads to an observed increase in the sensor effect in a composite sensor based on CeO_2_–In_2_O_3_ compared to the sensor effect in a single-component In_2_O_3_ layer.

Further enrichment of the composite with cerium oxide in the range 10 wt.% < X_Ce_ < 40 wt.% results in a sharp decrease in S_max_. The S_max_ value of the CeO_2_–In_2_O_3_ composite sensor containing 40 wt.% CeO_2_ is significantly less than S_max_ of pure In_2_O_3_. At the same time, the value of T_max_ decreases with an increase in X_Ce_ from 3 wt.% to 40 wt.% CeO_2_, regardless of how S_max_ changes.

With an increase in the concentration of CeO_2_ in the composite above 10 wt.%, the sensory response gradually decreases. This is due to the very low conductivity of systems based on CeO_2_ nanoparticles and their coagulation. As a result, larger CeO_2_ nanocrystals appear in the system, which leads to a significant decrease in the concentration of oxygen vacancies.

At CeO_2_ concentration >50 wt.%, the current flows through the crystals and the sensor response of the composite is approximately the same as in pure CeO_2_ and much less than in pure In_2_O_3_ [17]. This behavior is due to a decrease in the conductivity of the CeO_2_–In_2_O_3_ composite sensor with an increase in X_Ce_ above 40 wt.% and, as a result, the attainment of the conductivity level of pure CeO_2_. This means that with an increase in the content of cerium oxide above 10 wt.% and especially at X_Ce_ of more than 40 wt.%, the current flow paths through the conducting In_2_O_3_ crystals are interrupted and the current passes mainly through the CeO_2_ nanocrystals. Thus, the corresponding sensor response becomes as small as that of pure CeO_2_.

### 3.2. Impregnation of In_2_O_3_ Nanocrystals with Cerium Oxide

The liquid-phase method of impregnation allows the preparation of a composite in which a large number of nanoclusters are located on the surface of relatively large nanoparticles and in the near-surface layer. In processes such as impregnation, it is preferable to use cerium nitrate salts to obtain CeO_2_ nanoclusters of nearly uniform size [80].

The In_2_O_3_ nanopowder was kept in an aqueous solution of cerium nitrate for 24 h. This provided a uniform distribution of the salt solution on the surface and in the near-surface layer of the matrix oxide. After that, the water was evaporated at 70 °C and the remaining powder was heated in air at 450–500 °C for 3–5 h. As a result of such heat treatment, hydrolysis of the cerium salt occurs with the formation of Ce(OH)_4_ hydroxide and its subsequent dehydration to CeO_2_. The composite formed during impregnation represents a system consisting of initial In_2_O_3_ matrix oxide nanoparticles (50–70 nm in size), on the surface of which are small cerium oxide clusters no larger than 10 nm in size [12] (see inset in Figure 1).

The introduction of particles of such a small size into the composite should improve its sensor properties. First, the surface area on which sensor reactions occur is significantly increased. Second, the structural and electronic characteristics of metal oxide particles vary depending on their size. This change is especially pronounced for clusters smaller than 20 nm, which are often referred to as quantum dots [81]. An increase in the proportion of atoms present on the surface and in the near-surface layers of small clusters is accompanied by a weakening of the strength of their bonds in the lattice of small clusters compared to bonds in ordinary crystals. This leads to a significant decrease in the formation energy of vacancies, an increase in their concentration in the cluster lattice [82], and, accordingly, an increase in sensor activity.

In order to determine the structure and properties of the powdered materials obtained in this way, solid films were made from them. The impregnated powder was ground in an agate mortar with the addition of water. The resulting aqueous paste was applied to polycor plates and annealed at 550 °C until a constant resistance of the formed film was reached [12].

The addition of small amounts of CeO_2_ to In_2_O_3_ has only a marginal effect on the conductivity of the composite. Some increase in resistance observed in this case can be associated with electron transfer from conducting In_2_O_3_ crystals to CeO_2_ particles, since the work function from In_2_O_3_ (4.3 eV) is less than that from CeO_2_ (4.7 eV).

Based on XPS and EDS data, the cerium oxide present in the composite does not change the electronic structure of indium oxide. This finding indicates that during the formation of the composite, cerium ions do not penetrate into In_2_O_3_ crystals, but are deposited on their surface in the form of nanocrystallites or amorphous clusters. This is also indicated by the fact that in the composite containing 3 wt.% CeO_2_, cerium ions are absent in the surface layer of In_2_O_3_ crystals contacting with the CeO_2_ nanocluster.

The main obstacle to the incorporation into the In_2_O_3_ lattice and the substitution of indium atoms with a valence of +3 by cerium atoms (Ce^4+^) in this lattice is the difference in valence of these atoms. Calculations performed using the Reference Intensity Ratio (RIR) method [83] showed that up to 30% of cerium oxide in composites present in the nanocrystalline phase, probably in the form of small clusters, are not detected by XRD.

These cerium oxide nanoclusters contain a high concentration of oxygen vacancies, which are the centers of dissociative chemisorption of oxygen and hydrogen molecules [84,85]. Oxygen and hydrogen atoms are formed on the CeO_2_ nanoclusters as a result of the molecular dissociation. The spillover of these atoms onto the surface of In_2_O_3_ nanoparticles is accompanied by the capture of electrons by oxygen atoms from the bulk of the nanoparticles. Hydrogen interacts with the formed oxygen radical anions O^−^. The trapped electrons return to the conduction band of the metal oxide and increase the conductivity of the composite, and, consequently, increase the sensor effect. Such sensitization of the sensor effect by CeO_2_ nanoclusters occurs much more efficiently than the effect of relatively large CeO_2_ nanoparticles (see Section 3.1).

It is important to note that the addition of small amounts of cerium oxide leads not only to an increase in the maximum response of In_2_O_3_ to hydrogen, but also to a noticeable decrease in the temperature at which S_max_ is reached. Thus, the maximum response to 1000 ppm of hydrogen of a composite with 3 wt.% CeO_2_, which is observed at 265 °C, is approximately three times the maximum response of pure indium oxide, which is achieved at a higher temperature (320 °C) (Figure 1). Additives of cerium oxide also affect the dynamics of the sensor process. The response time of such a modified composite with the addition of hydrogen is within 1–2 s, the relaxation time of the sensor after removal of hydrogen from the system is ~120 s [12].

The T_max_ values for CeO_2_–In_2_O_3_ composites containing 3 and 10 wt.% CeO_2_ are 265 °C and 260 °C, respectively [12]. These values are 100 °C lower than for composites obtained by mixing nanocrystalline powders of cerium and indium oxides [17] (see Section 3.1), as well as for impregnated CeO_2_–SnO_2_ composites with the same content of cerium oxide [12].

Enrichment of composites with cerium dioxide up to 40 wt.% and more leads to a significant decrease in the sensor effect. Even at content of 15 wt.% in the composite, its sensor effect becomes the same as that of pure In_2_O_3_. The value of the maximum sensor effect to 1000 ppm of hydrogen for composite sensors containing 40 wt.% CeO_2_ does not exceed 10, which is more than seven times lower than the response of pure In_2_O_3_.

The sensor process leading to the afore-mentioned sensitization of the sensor effect by small additions of CeO_2_ clusters takes place at the interface between high specific surface area clusters and In_2_O_3_ nanocrystals [17]. Therefore, one of the reasons for the decrease in the sensor effect observed at a high concentration of CeO_2_ is probably the aggregation of these clusters, which occurs with an increase in the content of cerium oxide in the composite, and, as a consequence, a decrease in their specific surface area.

The sensor activity of porous CeO_2_–In_2_O_3_ nanotubes with respect to various gases depends on their composition and detection temperature. At low temperatures (25–110 °C), such a composite can effectively detect H_2_S; at higher temperatures, these nanotubes are an acetone detector. The In_75_Ce_25_ composite possesses optimal sensor activities. The maximum response of this composite to 20 ppm H_2_S at 80 °C is 498, and the highest response to 200 ppm acetone at 300 °C is 30. However, the dynamic performance of this composite is significantly inferior in both response time and recovery time to a sensor based on pure indium oxide [75].

### 3.3. Hydrothermal Method

To obtain CeO_2_–In_2_O_3_ composites by the hydrothermal method, we used chemically pure In(NO_3_)_3_·5H_2_O as indium oxide precursors, and Ce(NO_3_)_3_·6H_2_O to form CeO_2_. In these salts cerium ions are in valence states 3 and 4, respectively. The amount of cerium salt necessary for the formation of the CeO_2_–In_2_O_3_ composite of a certain composition was added to 0.34 mmol of In(NO_3_)_3_·5H_2_O and 4.2 mmol of urea. The resulting mixture was dissolved with vigorous stirring in 20 mL of 95% ethanol or 20 mL of bi-distilled water.

The solution thus prepared was placed in a Teflon-coated stainless-steel autoclave. The autoclave was closed tightly and kept at 160 °C for 5 h. After the autoclave was naturally cooled to room temperature, the formed precipitate was filtered off, washed several times with distilled water and absolute alcohol, and dried at 100 °C. The final product was annealed at 500 °C for 2 h.

The structure of the composites was determined by X-ray diffraction (XRD), transmission electron microscopy (TEM), energy dispersive X-ray spectroscopy (EDX), and X-ray photoelectron spectroscopy (XPS). The spectrum of pure indium oxide synthesized in ethanol in the absence of cerium additives exhibits both peaks from the main cubic c-In_2_O_3_ phase and from the metastable rhombohedral rh-In_2_O_3_ phase. The contents of these phases in the sample are 36.9 wt.% c-In_2_O_3_ and 63.1 wt.% rh-In_2_O_3_. When cerium is introduced into the composite, only the rhombohedral phase of indium oxide is observed in the spectrum; the cubic phase of In_2_O_3_ in the X-ray diffraction pattern is absent. In this case, a phase of cubic cerium oxide with the fluorite structure appears. Considering that the structure of CeO_2_–In_2_O_3_ composites depends on the method of their formation, in this study only CeO_2_–In_2_O_3_ composites obtained by the hydrothermal method from alcoholic solutions of indium and cerium nitrates are considered.

The application of the Rietveld method indicated that the content of the crystalline phase in the composite with 10 wt.% CeO_2_, within the error of the method, coincided with the total content of CeO_2_. This means that nearly all the cerium oxide is a separate phase, and only a very small fraction of CeO_2_ is dissolved in In_2_O_3_ nanocrystals. The particle size of cerium oxide in the composites containing 3 wt.% CeO_2_ is approximately 12 nm, while it is 10 nm in the composite with 10 wt.% CeO_2_. In this case, the particle size of indium oxide decreases by a factor of 3.5 compared to pure In_2_O_3_. Thus, from the TEM and HRTEM data, the composite consists of randomly arranged indium oxide nanoparticles of about 30 nm in size and cerium oxide nanoparticles ~10 nm in diameter.

**Figure 6:**
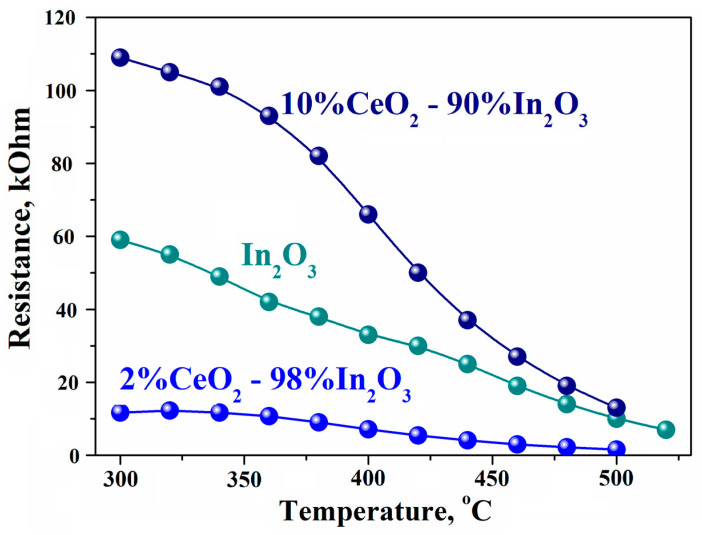
Temperature dependences of the resistance of In_2_O_3_ and CeO_2_–In_2_O_3_ composites.

At temperatures in the range of 300–520 °C, the resistance of CeO_2_–In_2_O_3_ composites containing less than 3 wt.% CeO_2_ is significantly lower than that of pure indium oxide (Figure 6). The small additions of CeO_2_ dissolved in the In_2_O_3_ lattice replace In^+3^ ions with Ce^+4^ ions. Such a substitution causes an increase in the concentration of conduction electrons. With a further increase in the content of CeO_2_, the substitutional solid solution becomes unstable, and CeO_2_ forms small clusters in the lattice with a higher work function than that of In_2_O_3_. The transition of electrons from In_2_O_3_ to the CeO_2_ clusters results in a decrease in the concentration of conduction electrons and, consequently, to the observed increase in resistance. Similar changes in conductivity depending on the composition of the composite were noted earlier in the study of structurally similar SnO_2_–In_2_O_3_ composites [34].

The sensor properties of hydrothermal CeO_2_–In_2_O_3_ composites were studied for composites based on the cubic modification of In_2_O_3_ [22,25,86]. In such systems, as well as in the composites obtained by impregnation considered above, at low concentrations (up to 10 wt.%) of cerium oxide, CeO_2_ additions lead to a noticeable increase in the sensor effect in the detection of reducing compounds, including hydrogen and CO. This result is attributed to the formation of a large number of defects in the In_2_O_3_ lattice upon the incorporation of CeO_2_ into it. The resulting defects include oxygen adsorption centers, specifically, oxygen vacancies. As a result, the concentration of chemisorbed oxygen in the composite increases, which is necessary for the detection of reducing agents in the air. At a content of about 3 mol.% cerium oxide these composites exhibit the highest sensitivity, good reproducibility, and long-term stability [87,88]. In the CeO_2_–In_2_O_3_ composites obtained by the hydrothermal method, the temperature T_max_, at which the sensitivity reaches its maximum value, also significantly decreases. It is worth noting that the decrease in T_max_ in these systems is more pronounced than for composites obtained by the impregnation method.

**Table 2:** Sensor response to various gases of composites with CeO_2_ additives.

Sensing Material	Synthesis Method	Sensor Response	References
3%CeO_2_–97%In_2_O_3_	Nanopowder mixture	87 at 2% H_2_, 18 at 0.46% CO	[17]
3%CeO_2_–97%In_2_O_3_	Impregnation method	205 at 1000 ppm H_2_	[12]
3%CeO_2_–97%In_2_O_3_	Hydrothermal method	33 at 9900 ppm H_2_, 5.6 at 970 ppm CO	This work
Ce-doped In_2_O_3_	Hydrothermal method	27.8 at 100 ppm ethanol, 63.4 at 100 ppm glycol	[22]
2%CeO_2_–98%In_2_O_3_	Hydrothermal method	20.65 at 50 ppm H_2_	[25]
Ce-doped In_2_O_3_	Hydrothermal method	27.8 at 100 ppm methanol	[89]
3%CeO_2_–97%In_2_O_3_	Hydrothermal method	41.8 at 200 ppm acetone	[90]
25%CeO_2_–75%In_2_O_3_	Electrospining method	498 at 20 ppm H_2_S	[75]
1%CeO_2_–99%SnO_2_	Impregnation method	20 at 1000 ppm H_2_	[12]
2%CeO_2_–98%SnO_2_	Chemical precipitation method	120 at 100 ppm ethanol	[24]
1%CeO_2_–99%SnO_2_	Sol-gel method	181 at 100 ppm butanone	[26]
5%CeO_2_–95%ZnO	Dip-coating method	75 at 100 ppm alcohol	[78]
1%CeO_2_–99%ZnO	Dip-coating method	23 at 100 ppm acetone	[78]

The value of T_max_ is determined by the competition between the sensor reactions of oxygen centers with the molecules of reducing agents and the processes of thermal desorption of the molecules of the reducing agent from the oxide surface [86]. Addition of catalytically active cerium oxide allows reduction of the activation energy of the sensor reaction and thereby increases the reaction rate and lowers the T_max_. In addition, the rate of recovery of the sensor to its original state after removal of the analyzed gas also increases. The sensor response and recovery times after detection of 200 ppm acetone at 250 °C are 2 s and 154 s, respectively, for a sample containing 3 mol.% CeO_2_, and 5 s and 182 s, respectively, for pure In_2_O_3_.

**Figure 7:**
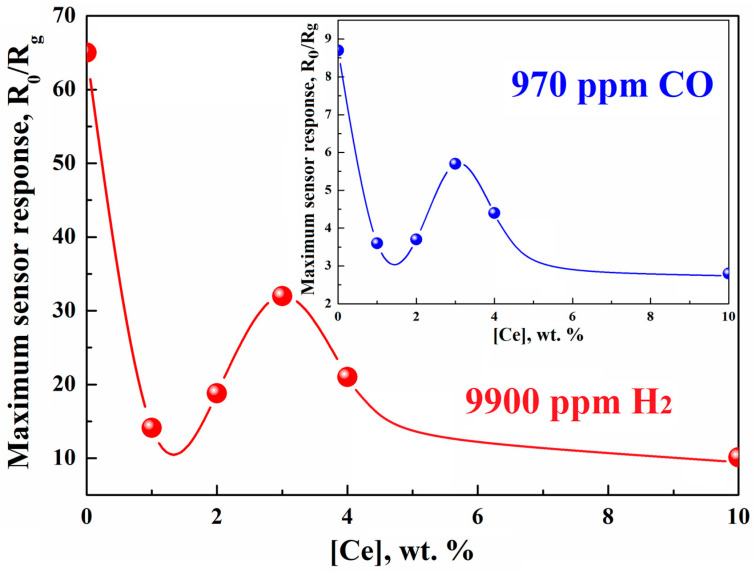
Concentration dependence of the maximum sensor response of the CeO_2_–In_2_O_3_ composites upon detection of 9900 ppm H_2_ (970 ppm CO in the inset).

**Figure 8:**
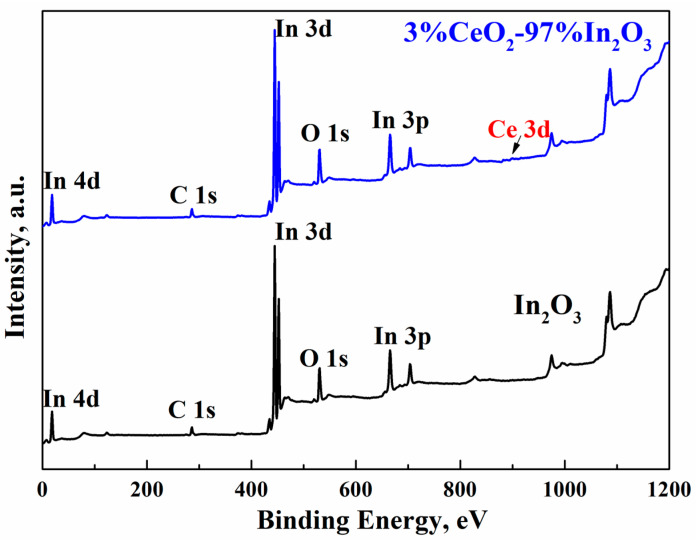
XPS spectra of pure indium oxide and 3%CeO_2_–97%In_2_O_3_ composite.

We studied CeO_2_–In_2_O_3_ hydrothermal composites based on the rh-In_2_O_3_, which were prepared using the method described above. The effect of introducing CeO_2_ into nanocrystalline In_2_O_3_ was found to change dramatically with a change in the phase state of In_2_O_3_ crystals. In the CeO_2_–In_2_O_3_ composites obtained based on the rhombohedral modification of In_2_O_3_ (rh-In_2_O_3_), we observed that CeO_2_ additions not only do not increase, but significantly reduce the sensory response to hydrogen and CO (Figure 7). To elucidate the features of the behavior of these composites in the detection of reducing compounds, we studied the XPS spectra of nanocrystalline films of pure In_2_O_3_ and composite films containing rh-In_2_O_3_ and CeO_2_ additives synthesized by the hydrothermal method (Figure 8). 

**Figure 9:**
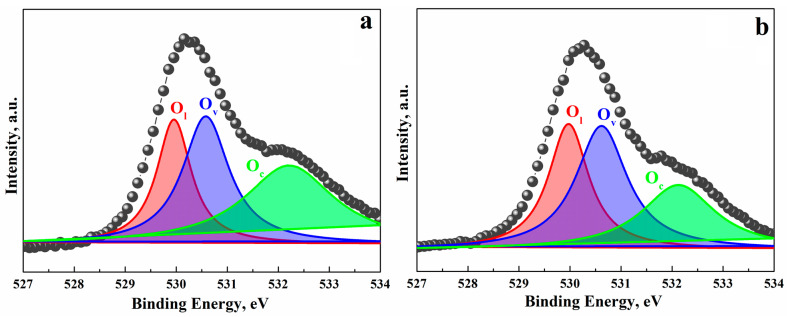
XPS spectra of O 1s (black circles) in In_2_O_3_ (**a**) and in the composite 3%CeO_2_–97%In_2_O_3_ (**b**).

Since the state of oxygen ions is of decisive significance for the sensor characteristics of the systems under study, we have analyzed the XPS O1s spectra. The asymmetric peak O1s can be divided into three peaks, which refer to three forms of oxygen: oxygen ions O_L_ in the crystal lattice; O_V_ ions contacting with oxygen vacancies in the lattice; and chemisorbed oxygen O_C_. The peak at the binding energy of 529.9 eV belongs to O_L_, the peak in the region of 530.6 eV, to O_V_, and the peak in the region of 532.3 eV to O_C_ [90] (Figure 9).

The measurements performed have shown that the content of oxygen vacancies in the composite is higher and that of chemisorbed oxygen is lower than in pure indium oxide crystals. Considering that the reactions of CO and H_2_ with chemisorbed oxygen determine the sensory response, the decrease in the concentration of chemisorbed oxygen when cerium oxide is added to indium oxide explains the drop in the sensor efficiency of the studied composites compared to the efficiency of nanocrystalline rh-In_2_O_3_.

The reasons why the inclusion of cerium oxide molecules or clusters in rhombohedral indium oxide crystals decrease the concentration of chemisorbed oxygen on the surface of these crystals are unclear and are currently being investigated. It should be noted that the chemical activity of an oxygen vacancy depends on its position in the lattice, which determines the environment of the vacancy.

**Figure 10:**
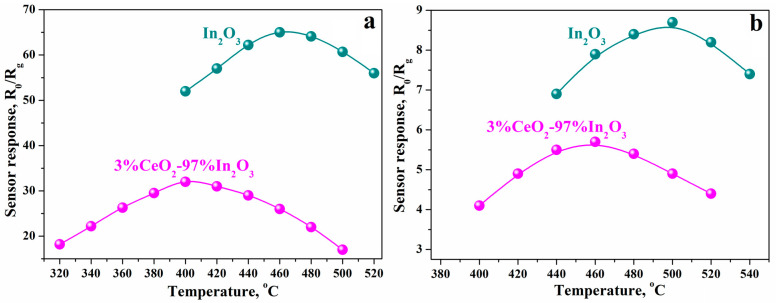
Temperature dependence of the sensor response of pure indium oxide and 3%CeO_2_–97%In_2_O_3_ composite during detection of: (**a**) 9900 ppm H_2_; (**b**) 970 ppm CO.

Characteristically, in CeO_2_ composites with rh-In_2_O_3_ synthesized by the hydrothermal method, as well as in composites based on cubic In_2_O_3_ (c-In_2_O_3_), the temperature at which the maximum response to H_2_ and CO is achieved decreases compared to T_max_ for pure rh-In_2_O_3_ (Figure 10). This shows that the change in the sensor sensitivity of the composites upon transition from the cubic to the rhombohedral phase of In_2_O_3_ is due not to chemical factors but to the morphological features of the composites. 

The sensitizing effect of dopants on the sensor properties of composites based on indium oxide obtained by the hydrothermal method is observed not only when using CeO_2_, but also some other metal oxides having the catalytic activity, in particular, zinc oxide (see, for example, paper [89]). 

In such composites containing up to 20% ZnO, no clear ZnO diffraction peaks were observed, which may be due to the incorporation of Zn ions into the indium oxide lattice or the formation of roentgenamorphic oxide phase. With an increase in the content of zinc oxide in the composite, the average size of indium oxide nanoparticles decreases from 25 to 9 nm. This once again confirms that Zn ions are introduced into the In_2_O_3_ lattice, since doping effectively inhibits the growth of nanocrystals. The particle size of zinc oxide is much larger than the size of In_2_O_3_, but as X_Zn_ increases from 20 to 85 wt.%, the particle size decreases from 72 to 25 nm.

Nickel-doped SnO_2_-based nanomaterials obtained by the hydrothermal method are also promising sensors for detection of various harmful gases [90]. Nickel-doped SnO_2_ nanomaterials have demonstrated very high sensor response, fast response and recovery times, and a lower operating temperature of 280 °C compared to 310 °C for pure SnO_2_. The responses of pure tin oxide and samples doped with ~3 mol.% nickel or zinc to 50 ppm CO at 280 °C were 4.50, 7.28, and 5.90, respectively. The higher sensor response to CO of the nickel-doped sample compared to pure SnO_2_ and zinc-doped SnO_2_ is due to the small size of crystallites, high specific surface area, and the formation of *n*-*p* heterojunctions between Ni and Sn.

## 4. Future Directions

Typically, various kinds of defects in the lattice of composite nanoparticles, which provide the formation of active centers on the surface of nanoparticles of the sensitive layer and gas adsorption on the particles, are considered independently of each other. In these studies, the links between the defects are not taken into account. Meanwhile, lattice defects interact with each other and form complexes. The effect of the defects interaction, in particular, complexes involving the metal ions and oxygen vacancies, can significantly increase the efficiency of such a sensor and change its sensitive characteristics. Therefore, one of the directions of our further research will be elucidation of the role of the interaction between defects of nanocrystalline particles in the detection process.

The interaction between nanoparticle lattice defects is only one of the possible types of interaction in the sensitive layer. Much more important are interactions between nanoparticles in mixed oxides (see Introduction and References [12,35]). Thus, for a broader program, the direction of our research will be related to the study of the role of various types of interaction between nanoparticles in the sensitivity and selectivity of semiconductor sensors.

## 5. Conclusions

The review considers the results of studies undertaken in the last 10–15 years and is devoted to elucidating the influence of the synthesis method and the structure of the nanocomposite on the properties of mixed semiconductor oxide sensitive layers during the detection of reducing gases in air. The role of active oxygen species and temperature in the detection process is analyzed. Various factors affecting the efficiency of sensors are discussed, specifically, the combination of oxides with different types of conductivity and the role of a catalytically active metal oxide.

The components of the sensitive layer can interact with each other. Various types of interaction of nanoparticles and their influence on the sensory process are considered. It is shown that such interactions can increase or decrease the amplitude of the response to the analyzed gases several fold. The material considered shows that, upon contact of particles with different work functions, new adsorption centers of predominantly polar molecules arise, which increase the efficiency of detection of such molecules. In this case, a redistribution of charges and the formation of defects, in particular, oxygen vacancies, occur in the crystal, which lead to strong changes in sensory effects.

A separate section is devoted to the comparison of the results of studies of the structure and sensitivity of two-component sensors consisting of identical metal oxides but prepared by different methods. The system comprising catalytically active (CeO_2_) and electron-rich (In_2_O_3_) nanoparticles was considered as an example. The following methods of preparing the sensitive layers were explored: mixing powders of different metal oxides, impregnation, and, in the last part of the article, the hydrothermal method. 

A comparative study of the temperature and composition dependencies of sensor properties for CeO_2_–In_2_O_3_ systems obtained by various methods was carried out. The study showed the following: (a) the maximum sensor response in the detection of H_2_ and CO gases is achieved for composites formed by the impregnation method; (b) composites obtained by all the methods investigated have exceptionally high response rates. The minimal response time does not exceed 1 s; (c) the minimal operating temperature, at which the composites have the maximum sensor efficiency, is obtained for the hydrothermal method. In this case, the optimum temperature is 50–100 °C lower than for composites obtained by impregnation or the mixing of commercial oxide powders.

## Figures and Tables

**Table 1:** Resistance and maximum sensor response of Co_3_O_4_–In_2_O_3_ composites to 1100 ppm H_2_ and 930 ppm CO.

Content of Co_3_O_4_ in the Composite, wt.%	Resistance at 215 °C, mOhm	Maximum Sensor Response to 1100 ppm H_2_	Maximum Sensor Response to 930 ppm CO
0	0.023	75	8.5
3	0.178	940	17.5
10	0.305	1324	21
20	5.7	538	18
40	13.2	310	17
60	17.1	36	4
80	0.320	7	3
100	0.014	10	3.5
